# Opening of the New Isolation Hospital, Acton

**Published:** 1905-01-28

**Authors:** 


### OPENING OF THE NEW ISOLATION HOSPITAL, ACTON.

The new Isolation Hospital at Acton was opened on the 18th inst. by Mr. Councillor Hunt, when a large number of visitors and ratepayers were present. Mr. J. W. Jarratt, the chairman of the Acton Urban District Council, took the chair, and opened the proceedings by addressing the company. Mr. F. A. Baldwin, chairman of the Works Committee, gave a description of the various buildings of the hospital, and then handed the keys of the hospital to the chairman of the Public Health and Isolation Hospital Committee, Mr. E. F. Hunt. Mr. D. J. Ebbetts, the surveyor to the council, then presented a memento of the occasion to Mr. Hunt, who, on the invitation of the chairman of the council, addressed those present, and declared the hospital duly opened. Mr. Corrie-Grant, M.P., then moved, and Mr. R. O. Davis, J.P., seconded, a vote of thanks to the council. The chairman of the council, in replying, invited the company to inspect the buildings. During the proceedings it was stated that a matron and staff of nurses were already engaged and were able to take up their work at once. The hospital, which can now receive patients, was formerly an old mansion known as "The Friars," situated in Wales Farm Road. The present building consists of an administrative block—which had formed the old mansion—an observation ward, a scarlet fever ward, a diphtheria and enteric fever ward; there are also a laundry, disinfecting plant, van sheds, and a mortuary. The grounds have been laid out with shady trees, so as to afford pleasure to convalescents during the hot months. Each ward has its own nurses' kitchen, and stands entirely by itself. The observation block contains four three-bedded rooms; the other wards each have seven beds. The floors are made of pitch-pine blockflooring. The hospital furniture was supplied by Messrs Shoolbred. The entire cost of the new hospital has been £15,000. Mr. D. J. Ebbetts was the architect, and Messrs. Hadley, of Staffordshire, the builders.

